# To shield or not to shield: shielding may have unintended effects on patient dose in CT

**DOI:** 10.1007/s00330-023-10211-3

**Published:** 2023-09-14

**Authors:** Heli Riitta Sinikka Larjava, Chibuzor T. M. Eneh, Hannele M. Niiniviita

**Affiliations:** 1 Department of Medical Physics, Turku University Hospital and University of Turku, Turku, Finland; 2 Department of Medical Physics, Turku University Hospital, Turku, Finland

**Keywords:** Computed tomography, Radiation dose, Phantom, Chest

## Abstract

**Objectives:**

The aim of the patient out-of-plane shield is to reduce the patient radiation dose. Its effect on tube current modulation was evaluated with the out-of-plane shield visible in the localizer but absent in the scan range in chest CT with different CT scanners.

**Methods:**

An anthropomorphic phantom was scanned with six different CT scanners from three different vendors. The chest was first scanned without any shielding, and then with the out-of-plane shield within the localizer but outside the imaged volume. All pitch values of each scanner were used. The tube current values with and without the out-of-plane shield were collected and used to evaluate the effect of overscanning and tube current modulation (TCM) on patient radiation dose.

**Results:**

The highest increase in cumulative mA was 217%, when the pitch was 1.531. The tube current value increased already 8.9 cm before the end of the scanned anatomy and the difference between the tube current of the last slices (with and without the out-of-plane shield in the localizer) was 976%.

**Conclusion:**

Applying an out-of-plane shield outside the scanned volume but visible in the localizer images may increase the patient dose considerably if the scanner’s TCM function is based only on localizer images.

**Clinical relevance statement:**

The use of an out-of-plane shield in CT may strongly increase the tube current modulation and thus provide the patient with a higher radiation dose.

**Key Points:**

*• Applying an out-of-plane shield outside the scanned volume but visible in the localizer images may increase patient radiation dose considerably.*

*• The effect is visible with scanners that use solely localizer-based tube current modulation.*

*• Features like overscanning may be difficult for the user to notice when planning the scanning, and yet they may affect tube current modulation and through it to patient dose.*

## Introduction

The number of examinations and the amount of cumulative patient radiation dose from computed tomography studies (CT) is increasing in Organisation for Economic Co-operation and Development (OECD) countries, due to their versatility in assessing various pathologies. CT exams provided about 50% of the collective effective dose from medical imaging in Europe and over 60% in the USA during the past decade [1–3]. Since CT examinations cause a considerable amount of medical radiation exposure, it is important to find methods to optimize the application.

The routine use of patient in- or out-of-plane shielding during x-ray-based imaging has received a lot of focus in recent years and continues to be a source of intensive debate. Concerns related to the use of contact shielding have led to a variety of publications with e.g., International Commission on Radiological Protection (ICRP) and International Atomic Energy Agency (IAEA) endorsing its use [4, 5], while others like the European as well as the American Association of Physicist in Medicine (AAPM) guidelines discourage its use [6, 7]. This mismatch between guidelines can also be seen in practice, because according to the survey by Johnston et al, there are differences in the use of patient contact shielding in clinical CT practice [8]. Patient contact shield, as defined by the British Institute of Radiology (BIR), means radiation protection applied directly to a patient undergoing diagnostic X-ray procedures, but it excludes shielding built into the imaging equipment or in the room design [9]. There are two types of contact shielding: bismuth shields placed on the patient and inside the imaged volume (in-plane shields), and lead shields wrapped around the patient, outside the imaged volume (out-of-plane shields). In this study, we focused on out-of-plane shields.

Helical scanning has a requirement to overscan the imaged volume to provide enough data to reconstruct the first and last image. The amount of overscanning can extend a considerable non-intuitive distance beyond the image volume as the overscanning in multidetector helical CT examinations can be from half to even two x-ray tube rotations [10–15]. The amount of overscanning depends on the manufacturer and scanner. In some scanners, there is a mechanical method to reduce the effect of overscanning (so-called adaptive or dynamic collimation). There is very little information of the exact amount of overscanning in the literature [10]. The length of overscanning is not visible before or during the scanning, thus making it impossible to take into account when placing an out-of-plane shield. The placement of an out-of-plane shield beyond the imaged volume is therefore not a simple and error-free task.

Tube current modulation (TCM) is a main technique to optimize the patient’s radiation dose in CT while keeping the image quality sufficient. Scanner modulates the tube current output in the *x*-, *y*-, and *z*-directions to maintain the predetermined image noise level over the complete image volume. TCM function uses one or two localizer images to measure the x-ray attenuation due to the patient anatomy (the localizer-based method, used by GE Healthcare and Canon) [10–14, 16]. Another method uses the same technique but it also measures the attenuation during scanning, updating the information from the previous 180° rotation and making corrections to the tube current accordingly (“near real-time modulation”). Siemens Healthineers and Philips Healthcare [16] use the latter method. Our hypothesis was that tube current increase when approaching the diaphgram is stronger using an out-of-plane shield than without.

## Materials and methods

**Fig. 1:**
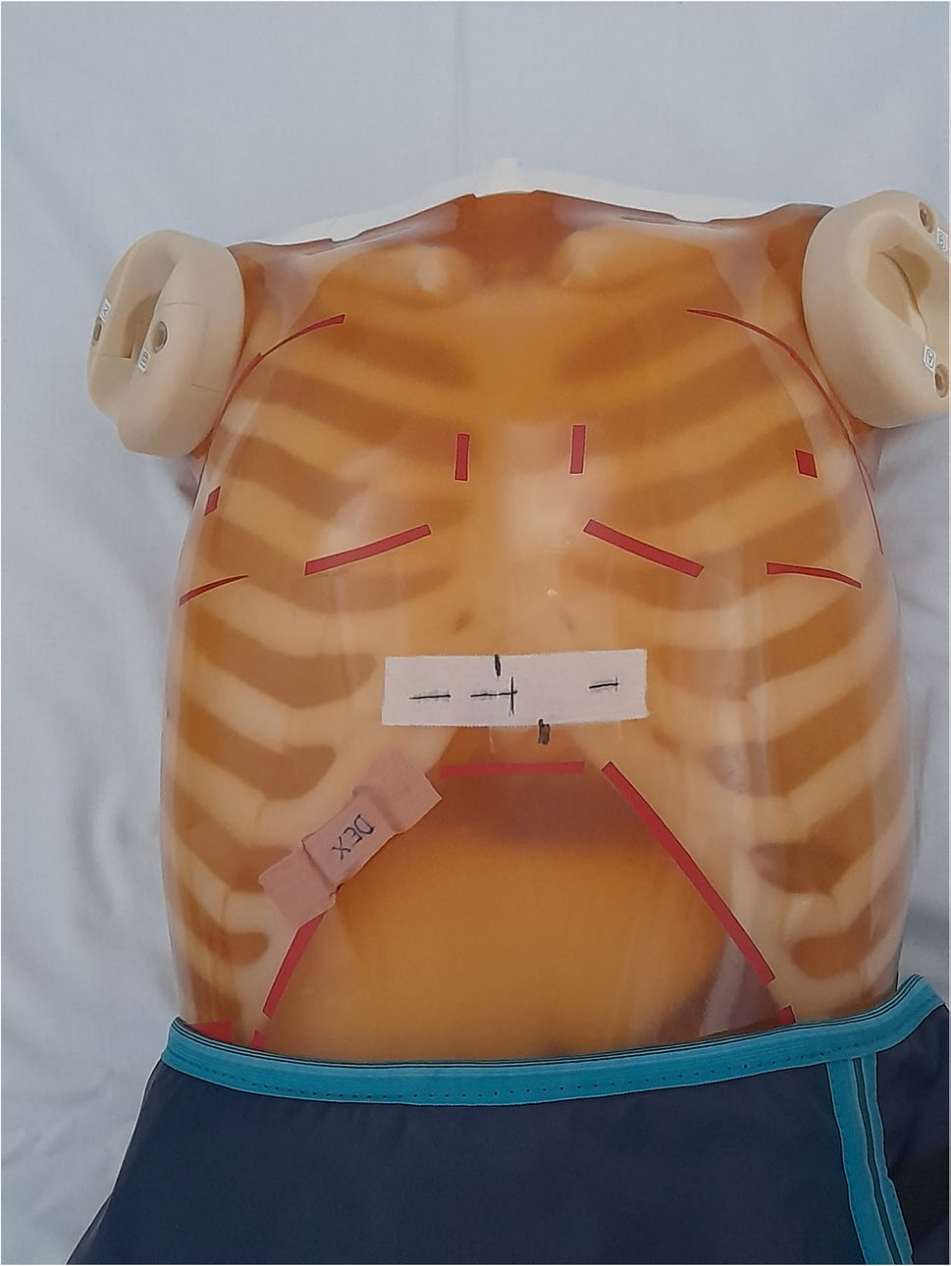
The contact shield placement 1 cm below the imaged volume on the Kyoto phantom. Shield was visible in localizer images but not included in the scanned volume

An anthropomorphic phantom and clinical helical thorax protocols were used to estimate the effect of patient contact shielding on TCM, with an out-of-plane shield (Scanflex Medical AB, 0.5 mm lead) placed to protect the abdomen and pelvis (Fig. 1). The phantom (Kyoto Kagaku, model PBU 60, Kyoto Kagaku Co Ltd) was scanned with six different scanners from three different vendors. The phantom corresponds to the average size of a small adult, 50 kg and 165 cm, and is made of tissue equivalent plastic. The CT scanners used were Optima 660 and Revolution (GE Healthcare); Aquilion One, Aquilion Prime, and Aquilion Prime SP (Canon Medical Systems Co, formerly known as Toshiba Medical Systems Co); and Siemens Somatom go.Up (Siemens Healthineers) (Table 1).

**Table 1:** Abbreviations, details of the clinical protocols of thorax scan, the used collimator width, and the amount of overscanning of the scanners used in this study. The source of information is included in the table

Scanner	Abbreviation	Collimator width (cm)	Amount of overscanning
GE Optima 660	GE Optima	4	Less than 1.2 rotations, calculated from [10]
GE Revolution	GE Revolution	8	Less than 1 rotation, calculated from [11]
Canon Aquilion One	Canon One	4	2 complete 360° rotations [12]
Canon Aquilion Prime	Canon Prime	4	2 complete 360° rotations [13]
Canon Aquilion Prime SP	Canon Prime SP	4	2 complete 360° rotations [14]
Siemens Somatom Go.Up	Siemens Go.Up	2.24	No information available

For every scanner, the available tube current range to be used in TCM was extended from the clinical protocol settings to begin from 10 mA and reach the maximum value allowed by the scanner, to better visualize the effect of shielding. Scans were repeated twice with all pitch values of each scanner (Table 1): first without any shielding wrapped around the phantom, and then with the contact shield visible in the localizer images but not included in the scanned volume. The beam collimation used in clinical protocols varied 2.24–8 cm. It was constant per scanner.

The phantom was scanned head first with standard clinical chest protocol from T1 to T12 vertebra, total length of scanned volume being 32 cm. The scan range was same for all scans. The shield was placed 1 cm outside the end of the scanned volume and wrapped around the phantom, beginning at the top edge of the 2nd lumbar vertebra and covering the abdomen and pelvis (Fig. 1).

An in-house developed MatLab program (The MathWorks, Inc.) was used to collect the average mA value of each slice to identify the location of the possible mA increase due to the out-of-plane shield visible in the localizer images. The cumulative tube current value of each scan was also calculated to determine the overall effect of the shielding.

When comparing the scans, the maximum limit of 20% mA difference between representative individual slices was set as acceptable. The value is selected according to AAPM report concerning the acceptability and reliability of computed tomography exposure reproducibility, stating that the maximum approvable variation from the mean value of measurements taken is 20% [17].

## Results

**Fig. 2:**
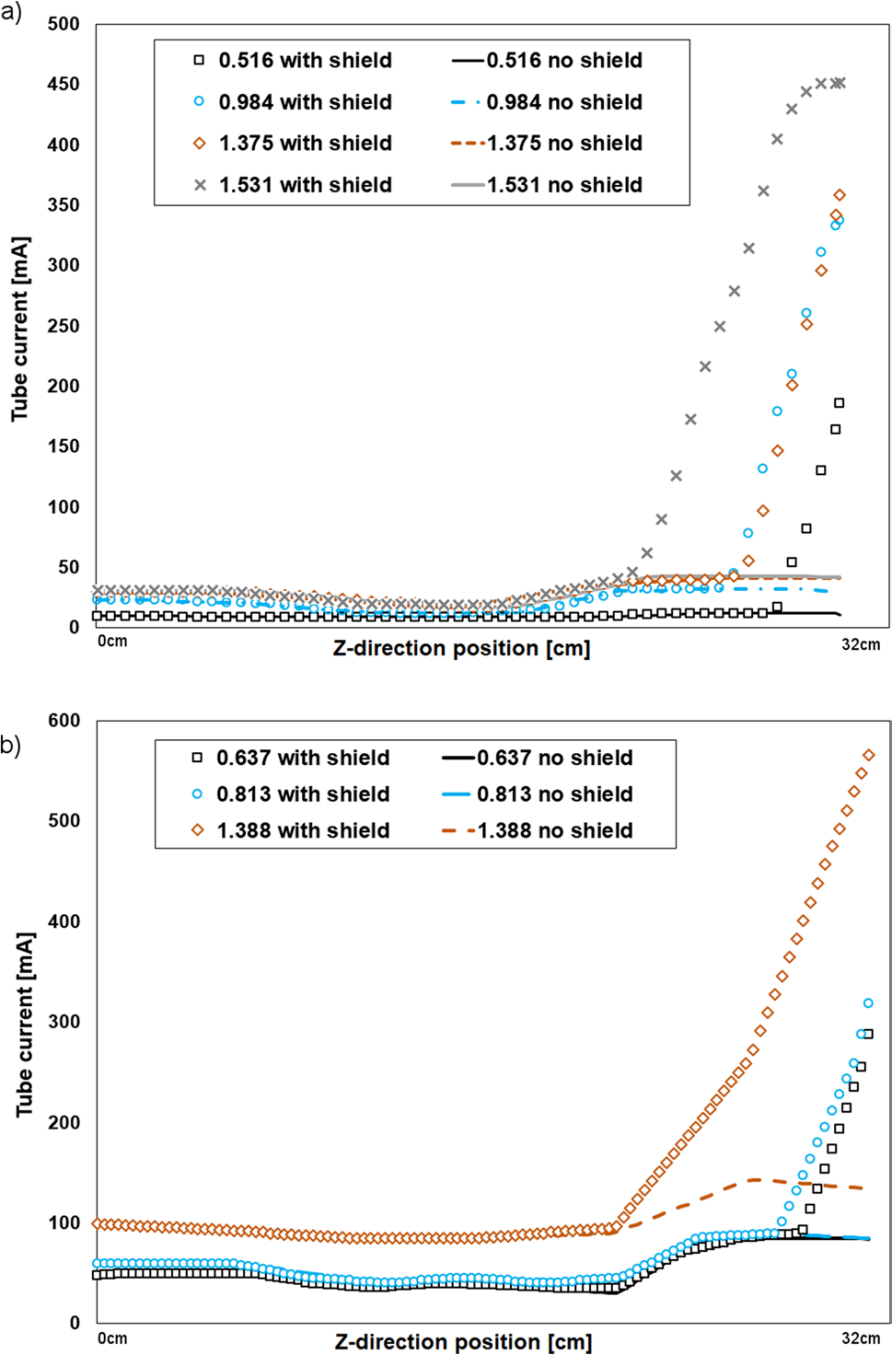
Tube current as a function of the slice, imaged using TCM and with all pitch values available at each scanner. Here we present the most notable differences in average mA values per image measured in our study. Location 0 cm represents a superior direction of a patient. (**a**) GE Optima 660, (**b**) Canon Aquilion Prime

For most scanners, we observed an increase in cumulative tube current when we used an out-of-plane shielding. In most cases, the effect was notable in scanners using only localizer-based TCM (GE and Canon scanners), but could not be seen in Siemens Go.Up, and was minimal with Canon Prime SP scanner when the pitch 1.388 was used. Visible shielding in localizer images appears to affect the mA modulation function of a scan, as does the pitch, also (Fig. 2).

**Fig. 3:**
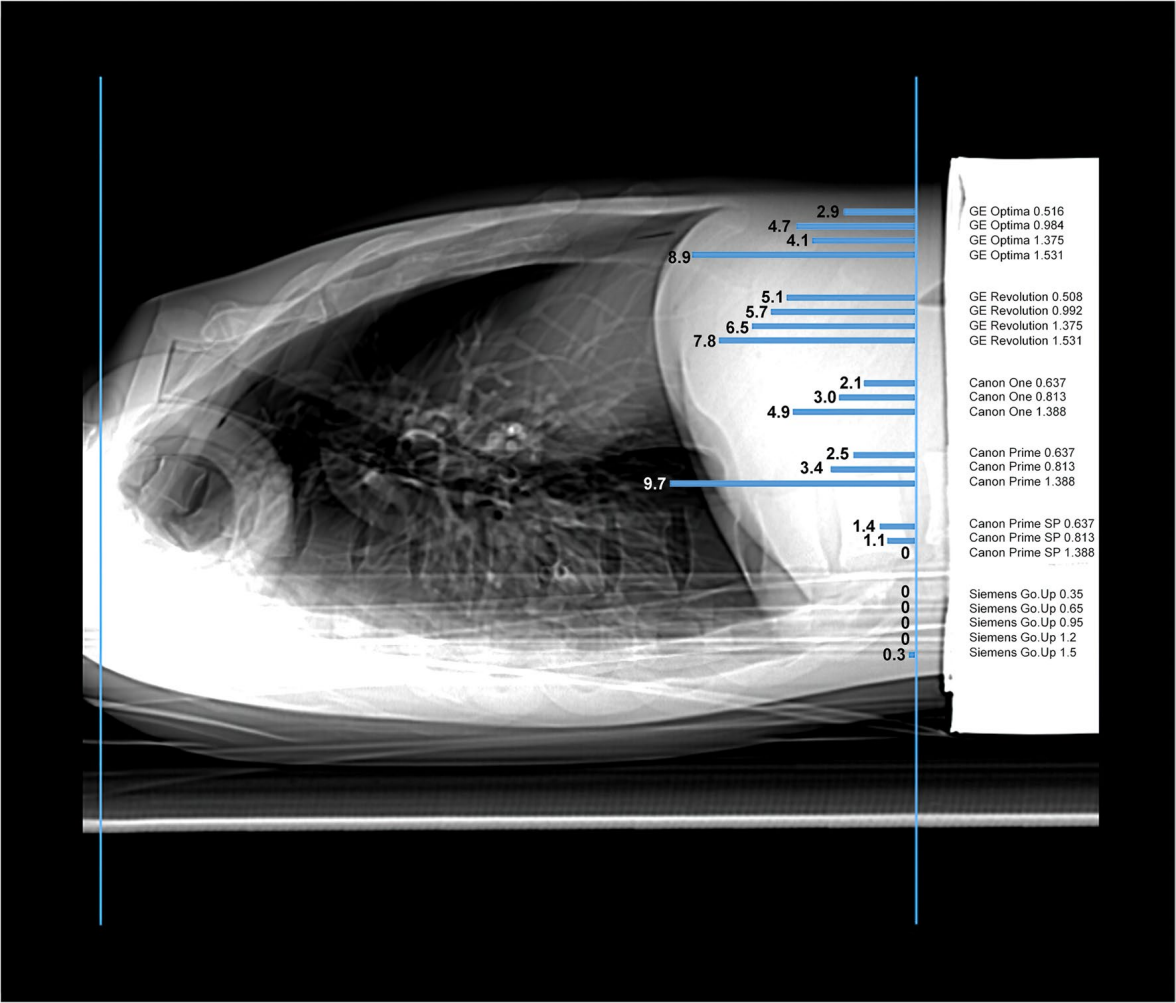
The tube current increase visualized over phantom anatomy. Blue horizontal lines represent the anatomical area in the phantom, which was exposed over 20% difference between the average tube current of the slice without and with an out-of-field shield in the localizer. The total length of the imaged volume was 32 cm (between vertical lines) and the earliest increase was detected 9.7 cm before the end of the scan

The earliest tube current increase was detected in Canon Prime, beginning 9.7 cm before the end of the scanned volume with 1.388 pitch (Fig. 2b, Fig. 3). In that incidence, the tube current difference of the last slices of the volume was 323%, and in the cumulative mA a difference of 53% (Table 2). The most notable difference with the cumulative mA value was, however, with another scanner. Anatomically, the second longest difference in single slices mA values was with GE Optima, being 8.9 cm with 1.531 pitch, and producing a 217% increase in cumulative tube current used in imagining the volume (Fig. 2a). This began 0.8 cm later in *z*-direction, but was remarkably steeper, as scanner imaged against its upper limit of TCM (Table 2). The tube current difference of the last slices was 976%.

**Table 2:** Cumulative tube current values for the chest scan and information whether the scan was done with or without out-of-plane shield in localizer images (but outside the scanned volume). The difference between these two values, their relative difference, and location of the > 20% tube current increase along the *z*-axis (starting point of the scan is valued as 0 cm and the end of the scan 32 cm)

	Pitch	Cumulative tube current, no shield (mA)	Cumulative tube current, with shield (mA)	Difference (shield-no shield) (mA)	Relative difference (%)	*z*-location of the > 20% tube current difference (cm)
GE Optima	0.516	5065	8808	3743	74	29.1
	0.984	11,302	24,004	12,702	112	27.3
	1.375	15,393	26,279	10,886	71	27.9
	1.531	15,818	50,218	34,400	217	23.1
GE Revolution	0.508	14,336	19,662	5326	37.2	26.9
	0.992	37,290	53,168	15,878	42.6	26.3
	1.375	53,312	71,015	17,703	33.2	25.5
	1.531	55,147	78,889	23,742	43.1	24.2
Canon One	0.637	63,954	66,499	2545	3.98	29.9
	0.813	69,547	76,739	7192	10.3	29.0
	1.388	121,837	143,648	21,811	17.9	27.1
Canon Prime	0.637	56,247	66,242	9995	17.8	29.5
	0.813	63,607	76,941	13,334	21.0	28.6
	1.388	109,280	167,543	58,263	53.3	22.3
Canon Prime SP	0.637	181,542	187,897	6355	3.50	30.6
	0.813	206,421	214,552	8131	3.94	30.9
	1.388	321,365	322,690	1325	0.41	-
Siemens Go.Up	0.35	14,934	15,065	131	0.88	-
	0.65	24,020	24,063	43	0.18	-
	0.95	36,181	36,230	49	0.14	-
	1.2	45,283	45,275	− 8	− 0.02	-
	1.5	57,605	57,402	− 203	− 0.35	31.7

## Discussion

We observed that using out-of-plane shielding (placing the contact shield within the localizer images but outside the scanned volume) is likely to increase the tube current in helical scanning with scanners using only localizer-based TCM method (in this study GE Healthcare and Canon Medical Systems Co). The effect seems to relate to pitch values since with larger pitch values used the image reconstruction algorithm requires more information from the area outside the primary interest anatomical volume, and the TCM algorithm tends to increase the tube current early to maintain sufficient image quality. With a scanner that applies a near real-time TCM (in this study Siemens Healthineers), the tube current increase was not detected, even if the out-of-plane shield was used and visible in the localizer image.

In similar research setting as ours, Begano et al observed that shielding increased the effective dose to mother as well as to the fetus, even with a scanner that corrects the TCM values based on the localizer images with the attenuation measurement of the previous half round of the x-ray tube [18]. However, in their research, the increase was due to the scattered radiation. Overscanning is a relevant factor when optimizing patient dose in CT. It differs prominently between vendors. With some scanners, the manufacturer-specific details of overscanning are difficult to find. The required overscanning can be even two rotations [10–15]. Thus, depending on beam collimation and pitch, the amount of overscanning can extend a considerable non-intuitive distance beyond the examination volume in the *z*-direction, amounting to or in some cases even exceeding 16 cm. We were not able to find from the literature the amount of overscanning for the Siemens scanner used in this study. When contacting the manufacturer, we received a reply stating that this information is not available. The latest found reference for the Siemens scanners is van der Molen and Geleijins’ study [15]. However, our results do give an impression that the amount of overscanning they have measured is not applicable in Siemens Somatom Go.Up scanner used in our study. With dose measurements conducted with a fixed tube current, scattered radiation follows an exponential relationship as a function of the distance from the edge of the scanned area, as also does the dose saved by patient contact shielding [19]. As Yu et al present, the dose saved with a use of a margin may be extremely small compared with the overall dose from the examination [20]. For scanners that only use a localizer-based TCM, our study indicates that it might be necessary to place the contact shield out of the overscanned area, which may extend even as far as a distance twice the width of the used beam collimation multiplied by the pitch from the primary scan plane edge. The dose reduction, if gained at all from the use of patient contact shield, may not outweigh the potential risk of artifacts. The TCM is only available in helical scanning. One might wish to use axial imaging to be able to apply an out-of-plane shield in some examinations. As Weber et al state, the use of axial acquisition allows the precise positioning of the out-of-plane shield. However, in clinical setting, the benefit of helical scanning is faster scan time, and using axial acquisition may result in motion artifacts [19].

In Fig. 2a, the tube current values are very similar between pitches 0.984 and 1.375. We were not able to identify the reason for that.

The difference in results between two Canon scanners (Prime and Prime SP) is notable. Canon Prime is an older model, and one can clearly see in the results the combined effect of the out-of-field shield, tube current modulation, pitch, and overscanning. With the newer model Prime SP, the tube current difference with and without shield is more obscure. It may be that the image reconstruction algorithm is of a new version, and better observes and utilizes the information of the active collimation. The slight difference in Prime SP results with pitches 0.637 and 0.813, and with pitch 1.388, may be due to the x-ray tube placement in respect to couch.

If adaptive or dynamic shielding is not implemented in the scanner, an out-of-plane shielding could reduce radiation exposure due to overscanning. Unfortunately, the TCM algorithm uses data from the localizer outside the imaged volume that increases the mA although this may not be necessary for constant image quality. The impact of this mA increase on a constant image quality was not examined in this study. The clinical protocols use a lower maximum mA level for TCM. Therefore, the overscanning effect on TCM might in clinical scanning be lower as in some cases the upper mA limit for TCM was reached (see Fig. 2a, pitch 1.531).

There are limitations in this study. We could have measured the dose more exactly with thermo- or radiophotoluminescent dosimeters, instead of using the tube current value indicated by the scanner. However, in this context, tube current is directly proportional to the dose, and thus applicable when evaluating the effect of an out-of-plane shield to patient dose. Using such dosimeters would also have enabled us to measure the effect of adaptive or dynamic collimation, which we now were not able to do. There may be some fluctuation with the placement of the contact shield due to the anatomical shape of the phantom, but the effect on the results is assumed to be small. The beam collimation used in clinical protocols varied 2.24–8 cm. Since we compared the scanner properties for each scanner, not between scanners, we considered it not essential. The differences in the TCM due to the x-ray tube placement in respect to couch, in the beginning of the scan, would have been less meaningful if we had repeated the scans multiple times.

Some user manuals provided the scanner-specific amount to overscan, but for few scanners the information was not found (Table 1). Since we did not measure the exact dimension of overscanning, it has only been reported here as an additional information. However, the results we observed are unambiguous. While the results of this study are limited to phantom scans using chest protocols, they are likely applicable to scans of other anatomy, too.

In the light of our results, we recommend not to apply an out-of-plane shield for helical CT thorax scans if using a scanner that applies only the localizer-based TCM.

## Abbreviations

AAPM: American Association of Physicist in Medicine
BIR: British Institute of Radiology
IAEA: International Atomic Energy Agency
ICRP: International Commission on Radiological Protection
OECD: Organisation for Economic Co-operation and Development
TCM: Tube current modulation

## Declarations

### Guarantor

The scientific guarantor of this publication is Jani Saunavaara, Adjunct Professor, Department of Clinical Medicine, Institute of Biomedicine in University of Turku.

### Statistics and biometry

No complex statistical methods were necessary for this paper.

### Study subjects or cohorts overlap

No study subjects or cohorts have been previously reported.

### Methodology

• Experimental

• Performed at one institution
